# Diphtheria: How Is It Propagated?1Read before the Medical Society of the County of Monroe, June 6, 1894.

**Published:** 1894-08

**Authors:** G. W. Goler

**Affiliations:** Rochester, N. Y., Medical Inspector, Rochester Health Department; 54 South Fitzhugh Street


### Original Communications.

### DIPHTHERIA: HOW IS IT PROPAGATED?[1]

By G. W. GOLER, M. D., Rochester, N. Y.,
Medical Inspector, Rochester Health Department.

In this paper I intend to confine myself to a discussion of those means which seem to operate in propagating diphtheria.

In a report to the health board of this city, based upon an examination of the age, sex, school, occupation, milk supply and sanitary conditions of the dwelling of each of 100 patients with diphtheria, the following causes were found to be mainly responsible for the spread of this disease: (1) Gross defects in the plumbing and drainage (both house and subsoil) within and beneath the house. (2) Contact, either direct or indirect. (3) Contact through the milk supply.

One hundred cases of diphtheria were reported in seventy-four dwellings with a mortality of twenty-three. Fifty-one, or 69 per cent., of these seventy-four dwellings were in bad sanitary condition, in eighteen, or 23 per cent., no gross defects could be found and in five, or 6.5 per cent., the illness was found to have arisen after contact with persons infected with the disease.

Diphtheria has long been associated with bad sanitary surroundings, open sewers, defective drains, water closets and urinals, within or near dwellings, because, as has lately been demonstrated,

1. Read before the Medical Society of the County of Monroe, June 6, 1894.

in the albuminoid refuse which collects about such places bacillus diphtheria finds food, heat and moisture essential to its growth and multiplication. When to bad household sanitation is added the inadequate provision for subsoil drainage that obtains in many of our urban dwellings, we have those conditions which predispose to diphtheria by lowering the vitality of persons who find homes in such dwellings, so producing a favorable soil upon which diphtheria bacilli may be received and grow.

A causative relation between diphtheria and gross sanitary defects about dwellings has not until recently been shown to exist. Statistics from reports of the Local Government Board's health officer at London, in 1891, show that of fifty different epidemics of diphtheria in England, only four could be traced to direct contagion. The rest were all connected with foul cess-pools, deficient drainage and sewerage, and the proximity of dirty animals and decomposing organic matter.

Convincing evidence of the relation between diphtheria and unsanitary surroundings could not then be furnished, because, until lately, positive experimental proof was lacking that bacillus diphtheria occurred in or about houses where diphtheria was found. Recently L. Fisher, of New York, made cultures from eighty-five traps in tenement houses; forty-five had to be eliminated because of accident, leaving forty examinations entirely reliable. Twenty of these were negative, twelve yielded pathogenic bacteria, eight more pathogenic microörganisms, chiefly feces bacilli, which could not be differentiated. Some of the pathogenic cultures yielded diphtheria bacilli.

The results of these experiments in discovering diphtheria bacilli in a great number of cases are not such as to awaken intense enthusiasm. The work, however, must be looked upon as a good beginning in a novel field of investigation, the results of which will doubtless show just what part bad plumbing may play in spreading diphtheria.

A second influence that contributes to the spread of diphtheria is contact, either directly with a patient in an atmosphere more or less charged with contagion, or indirectly by means of anything containing diphtheria bacilli—table utensils, milk, drinking cups, pencils, etc., that might be conveyed to the mouth. Clothing, bed linen, articles of furniture, on which germ-laden dust may have lodged, all remain sources of danger for weeks or months after an illness from diphtheria has terminated. The disease has been traced to an old swab which was laid away in a bureau drawer. Several years before the swab had been used upon the throat of a child that died of diphtheria.

In a European town diphtheria became epidemic after the bodies of several persons who died of diphtheria had been exhumed for reburial. Other cases might be cited where diphtheria has been traced to indirect contact, but all sources of error were not eliminated and all lack the weight of bacteriological proof in their support.

A part of this question of contact which most interests us is this : When a patient apparently recovers from diphtheria, when he has been newly bathed, his clothing and bed linen thoroughly cleaned, aired and fumigated after the most approved plan, how long after this period is he capable of transmitting the disease to others ? Four years ago Klein, in England, Roux and Yersn, in France, found diphtheria bacilli in the throats of patients from a few days to two weeks after recovery was thought to have been complete. The New York City board of health, through its pathologist, Dr. Herman Biggs, took up this line of investigation last year. Four hundred and fifty cases of diphtheria were subjected to frequent bacteriological examination. In 245 of these 450 cases the diphtheria bacilli disappeared within three days after complete separation of the false membrane ; in 160 cases the diphtheria bacilli persisted for a longer time—namely, in 103 cases for seven days, in thirty-four cases for twelve days, in sixteen cases for fifteen days, in four cases for three weeks, and in three cases for five weeks after the time when the exudation had completely disappeared from the upper air-passages.

Dr. Biggs says :

> In many of these cases the patients were apparently well many days before the infectious agent had disappeared from the throat. These results show that in a considerable proportion of cases, persons who have had diphtheria continue to carry the germs of the disease in their throats for many days after all signs of the disease have disappeared. No doubt the disease is largely disseminated by these persons who are apparently well and who mingle with others while their throat secretions still contain the diphtheria bacilli.

These are significant facts. They call for a reorganization of our quarantine and disinfection regulations and the substitution of laws for the prevention of diphtheria similar to those now in force in New York City. The New York board of health has adopted a rule that no person shall be considered free from diphtheria until a bacteriological examination shall have proven the absence of diphtheria bacilli from the throat. Until by means of such an examination the throat is proven to be free from diphtheria bacilli, quarantine will be enforced and disinfection will not be performed by the department.

These experiments and the action of the New York board have an important bearing upon the occurrence of diphtheria among school children. If, as has been found by careful investigations, diphtheria bacilli may and often do persist for days after all membrane has disappeared from the throat, we can understand how children returning to school soon after recovering from diphtheria may carry the germs, not alone in their clothing, but in their throats as well. School children carry so many foreign substances to their lips—pencils, pens, paper wads, drinking cups, etc., that in this new light it is not difficult to understand how contagion may be transmitted from child to child. Thus, in a school district where diphtheria breaks out, a number of children may become carriers of the disease, even though quarantine has been strictly enforced for the prescribed length of time and disinfection carried out according to the rules of health departments in most cities.

To show how diphtheria may be carried among school children and the subtile means by which it may be transmitted, the following is deemed important: Twenty-three households in one primary school district have been scourged by diphtheria during the present school year, while in a portion of the city immediately adjoining the district and three times larger, the number of cases was not so great. The disease began to appear last September in two portions of the district where the sanitary conditions were wretchedly defective, open sewers and cess-pools in front of the houses, badly drained cellars. These defects were remedied and the disease was lulled for a time, with now and then an occasional outbreak among children of school age. The school building that these children attended was found to be in excellent sanitary condition, the main sewerage of the school and the whole district was without gross faults, so far as could be discovered. Contagion through milk or ice could not be traced. Only two known causes remained open for investigation, and these were contact at home or at school and unsanitary conditions about the dwelling.

Some of the dwellings were found in bad sanitary condition, but when taken altogether the gross defects in plumbing and drainage did not seem to furnish a sufficient cause for the spread of the disease. It was believed that some children had returned to school before complete recovery from diphtheria, and had thus served to communicate the disease to others. Inquiry developed the fact that a large number of children that became ill, belonged to one school. Across the hall from this room there is a faucet and several tin cups from which all the children drink. During a visit made to the school building, in company with Prof. Dodge, two of those cups were taken by him for bacteriological examination, and both cups were found to yield cultures of diphtheria bacilli from the foreign material about their rims. Here, then, we have proof of the possibility of propagating diphtheria by means of drinking cups, and, if this is so, why not other diseases as well? It is probable that children had entered school after apparent, but not real recovery from diphtheria, and, drinking from public cups, transmitted diphtheria germs to the cups from which they were deposited upon the lips of others who drank therefrom.

Another danger that menaces school children lies in the common practice of distributing lead pencils so that on one day a child gets one pencil, and on another day a pencil that may have been used by some child with a contagious disease.

Third, the relation of milk supply to diphtheria.

In 1878, W. H. Power, of London, to whom belongs the credit of first tracing the relation of milk supply to diphtheria, in a report submitted conclusive evidence that the milk supplied to certain families had been the means of spreading diphtheria among them. Several epidemics of diphtheria in different parts of England were by him traced to the milk supply, after every other source of contagion had been eliminated.

How was the contagion carried to the milk?

Was it introduced in the process of handling, or did it come from the cows?

Both of these questions may be answered in the affirmative. In the manure and filth near cow stables diphtheria and other pathogenic bacteria find necessary food, heat and moisture for their rapid multiplication. Milk also furnishes an excellent medium for bacterial growth, and from the reeking floors and adjoining dung heap to the bodies and udders of the cows and so into the milk pail, is a distance over which microörganisms may travel with facility. A visitor at certain cow stables could not fail to observe crusts of manure upon the bodies and udders of many of the cows. If the milking process were watched, fine manure dust might be seen falling into the milk pails, carrying with it many bacteria more or less capable of doing harm. When milk is cooled in or near cow sheds, or other places where dust has more or less free access to it, numerous bacteria are carried into the milk and there await a proper temperature to multiply and a fertile soil in which to produce their characteristic diseases. If diphtheria occur in the milkman's family, diphtheria bacilli may find their way into the milk. In this city an investigation of the milk supplied to families in which diphtheria was reported, showed among other cases the following: One milkman having stables in an adjoining town, distributed milk to seven families in which one or more cases of diphtheria broke out. There were three deaths. This milkman had three cases of diphtheria in his own family, and, during the illness in his own family, milk from his stables was being sold in the city. Further inquiry showed that diphtheria did not appear in the families of his customers until after the disease had affected this milkman's family. Other cases might be cited in which there is good reason to believe that diphtheria has been carried in milk, but they lack the support of bacteriological proof.

Between diphtheria in man and diphtheria in cows an exact relation has been proven, both by observation and experiment. To a beginning in this work we are indebted to Powers. So early as 1878, while investigating an epidemic of diphtheria involving 573 households, he, by a process of exclusion, was led to believe that actual cow conditions had brought about the results observed. Upon the examination of the cows of two large dairies from which much milk was obtained in the infected district, Mr. Powers was able to find ulcerations upon the udders and teats of the cows, and also to find some of them in poor health. Satisfied that the source of the epidemic had been found, he embodied the results of his finding in a report to the government and also presented a paper to the London Pathological Society in 1879, in which he maintained that a similar relation existed between bovine diphtheria and human diphtheria as between tuberculosis and anthrax.

In several other epidemics in England, Powers was able to prove the existence of diphtheria in cattle by the strongest circumstantial evidence. Previous to 1883, and until 1889, the Klebs-Loeffler bacillus was not universally recognized as the cause of diphtheria. So that in Powers' earlier observations there was no known way of demonstrating their truth or falsity. But in 1889, Klein in a paper submitted the results of his experiments upon milch cows, in which he showed by culture tests that true diphtheria may exist in these animals. Cats fed upon milk from inoculated cows showed all the symptoms of diphtheria, one animal dying of the disease. Thus, ten years after Powers' discovery, his observations were confirmed by experimental proof.

Diphtheria is not a widespread disease among cattle. Inquiry among local owners of milch cows leads to the belief that few cases of manifest disease of the milk apparatus are seen. It was found that most men had seen cows with severe disease of the teats, some of which were attended by sloughing and loss of the entire nipple. During the illness the cows always suffered in health.

It is only within the scope of this short paper to briefly point out remedies applicable under those conditions which have been shown to exist. These are:

(1) Repair defective plumbing and secure adequate drainage in every home where defects are found to exist.

(2) Guard all means of contact by sufficient and rigid quarantine, so that patients having had diphtheria shall be really well before they are allowed to mingle with others. Public drinking cups in schools and elsewhere to be abolished. A system of distributing lead pencils in public schools to be so arranged that each child shall have his own lead pencil.

(3) Laws governing the construction and maintenance of those stables from which our milk supply is drawn.

(4) The sterilization of all milk for children's use.

54 South Fitzhugh Street.

